# Comparative Evaluation of Camelina Seed Oils Obtained by Cold-Pressing and Solvent Extraction

**DOI:** 10.3390/foods13223605

**Published:** 2024-11-11

**Authors:** Slađana Rakita, Nedeljka Spasevski, Ivan Savić, Ivana Savić Gajić, Jasmina Lazarević, Danka Dragojlović, Olivera Đuragić

**Affiliations:** 1 Institute of Food Technology, University of Novi Sad, Bulevar Cara Lazara 1, 21000 Novi Sad, Serbia; nedeljka.spasevski@fins.uns.ac.rs (N.S.); jasmina.lazarevic@fins.uns.ac.rs (J.L.); danka.dragojlovic@fins.uns.ac.rs (D.D.); olivera.djuragic@fins.uns.ac.rs (O.Đ.); 2 Faculty of Technology in Leskovac, University of Niš, Bulevar Oslobodjenja 124, 16000 Leskovac, Serbia; savicivan@tf.ni.ac.rs (I.S.); savicivana@tf.ni.ac.rs (I.S.G.)

**Keywords:** camelina seed, oil, fatty acids, antioxidants, oxidative stability, cold-pressing

## Abstract

This study aimed to analyze the physicochemical properties and nutritional quality of oil extracted from the camelina seed genotypes NS Zlatka and NS Slatka, grown in Serbia, using both Soxhlet extraction with *n*-hexane and the cold-pressing technique. Extraction technique did not have an effect on oil yield. Camelina oils exhibited satisfactory physicochemical characteristics, which were influenced by the extraction methods. The oils were rich in polyunsaturated fatty acids, with α-linolenic acid being the most abundant. They were characterized by a balanced ω-6 to ω-3 ratio (0.5), low atherogenicity index and thrombogenicity index values, and a relatively high hypocholesterolemic/hypercholesterolemic ratio. Cold-pressed oils contained significantly higher amounts of α- and γ-tocopherols and showed greater oxidative stability at moderate temperatures, as confirmed by the Schaal oven test. Despite this, their oxidative stability decreased at elevated temperatures (Rancimat test) compared to solvent-extracted oils. Conversely, solvent-extracted oils had higher levels of β-carotene and showed superior resistance to high-temperature conditions. Due to its unique characteristics, nutritional properties, and health-promoting attributes, cold-pressed camelina oil presents significant potential for application in food, nutraceutical, feed, and cosmetic industries.

## 1. Introduction

Camelina (*Camelina sativa* L.) is an agricultural oil plant species from the Brassicaceae family. In the Balkans, it is also known by the name wild flax, podlanak, and Siberian flax. Camelina has modest agronomic growing requirements; it efficiently takes in water and nutrients from the soil and does not have a high demand for nitrogen. For this reason, it is often grown on low-quality lands. It is especially resilient in dry regions and in comparatively low-fertility or salty soils. The simple growing conditions, the rich chemical composition of the oil, and the wide range of possible applications make this plant species increasingly attractive worldwide [1]. Consequently, intensive efforts are being made to develop new camelina genotypes, including the spring genotypes NS Zlatka and NS Slatka, which are specifically adapted to the climatic conditions in Serbia [2]. Camelina seeds are rich in vegetable oil (30–50%), which has a high nutritional value, owing to a high percentage of polyunsaturated fatty acids (PUFAs) (40–60%). Most of them belong to ω-3 fatty acid (α-linolenic acid), which represents 35–40% of the total amount of fatty acids. This oil also contains a significant amount (15–18%) of ω-6 linoleic acid and more than 30% monounsaturated fatty acids (MUFAs) [3]. The high percentage of unsaturation makes the oil suitable for the production of polyols [4]. It is also considered a promising raw material for the production of biodiesel [5]. Camelina oil is known to contain substantial levels of tocopherols, phytosterols, and phenolic compounds [6]. The presence of these compounds makes the oil stable and resistant to oxidation. It is versatile and used in various fields, such as the food industry [6,7], cosmetics, pharmaceuticals, animal feed [8], and the production of adhesives, lubricants, and coatings [9]. Furthermore, due to its exceptionally high nutritional value, camelina oil can significantly enhance human and animal health when included in the diet [10,11].

Recent trends in oil extraction have emphasized replacing harmful organic solvents with innovative green and renewable alternatives to create safer, higher-quality products [12]. There has been a growing focus on the safety, environmental impact, and cost-effectiveness of extraction methods. Although modern extraction techniques (microwave-assisted enzymatic extraction, ultrasound-assisted extraction, supercritical fluid technology, high pressure-assisted extraction, and pulse electric field-assisted extraction) are superior from an economic and ecological standpoint, they are still only being analyzed in laboratory settings and have not yet been widely adopted for commercial use. The expensive equipment represents one of the disadvantages of their application [13]. The cold-pressing technique [3] and Soxhlet extraction with *n*-hexane [14] are commonly employed for the oil extraction from camelina seeds. The cold-pressing method extracts oil at low temperatures (generally below 45 °C) without using solvents [15] and is regarded as a simple, cost-effective, and, most importantly, environmentally friendly method. While cold-pressed oil is considered high in quality, its lower yield often diminishes the advantages of its superior quality. In contrast, hexane extraction produces higher yields, but the presence of solvent residues in the final product can pose health risks. Additionally, hexane’s flammability and the need for an evaporation step to remove the solvent from the oil make this extraction method less desirable [16]. Usually in the industry, solvent extraction requires refining and processing before the finished product is obtained [17]. The primary goal of refining is to eliminate unwanted components from crude oil, resulting in a high-quality product that is acceptable to consumers. The oil industry primarily utilizes two main processes: chemical refining and physical refining. Each of these processes includes various stages, such as degumming, neutralization, bleaching, deodorization, and winterization. Cold-pressed oils retain all the naturally occurring bioactive compounds from the seeds, including essential fatty acids, phenolic compounds, flavonoids, phytosterols, carotenoids, and tocopherols, making their sensory and aromatic qualities key factors in consumer acceptance, beyond considerations of availability, safety, and price. In particular, the volatile aromatic compounds in cold-pressed oils have been found to significantly impact their overall quality and consumer appeal [3,18].

Numerous research studies have focused on evaluating the quality attributes and oxidative stability of camelina oil obtained by cold-pressing [11,14,19,20,21,22,23,24]. Other studies have examined the physicochemical properties, antioxidant potential, and fatty acid and tocopherol composition of camelina oil obtained by *n*-hexane extraction [14,25]. However, most published studies to date have evaluated only a single extraction method, limiting direct comparisons of camelina oil quality across different extraction techniques. Belayneh et al. [16] compared super critical CO_2_ extraction, cold-pressing, and hexane extraction of camelina oil, focusing on oil yield and composition (fatty acids, tocopherols, and phytosterols). They found that the extraction method did not significantly affect the fatty acid profile or tocopherol levels but did influence oil yield and phytosterol content, with cold-pressing yielding the lowest and supercritical CO_2_ and hexane extractions producing higher levels. Moslavac et al. [26] investigated the effect of the oil extraction process from camelina seeds, which involved screw-pressing followed by extraction with supercritical CO_2_, on oil quality. Although supercritical CO_2_ extraction is considered an alternative “green” extraction method, it requires specialized equipment and is generally more costly [26]. As mentioned earlier, each extraction method has advantages and disadvantages, and some methods may be more suitable depending on the oil’s intended application and desired quality characteristics.

This study aimed to evaluate the influence of solvent extraction, commonly used in industrial contexts for its high yield efficiency, and cold-pressing, a solvent-free method suitable for on-farm applications, on the physicochemical properties, oil composition, and oxidative stability of oils obtained from two Serbian camelina seed genotypes. Our findings are expected to contribute valuable insights for camelina seed producers looking to optimize oil quality based on available resources and production scales, thereby advancing the versatility and market potential of camelina oil.

## 2. Materials and Methods

### 2.1. Chemicals and Reagents

In this study, 96% (*v*/*v*) ethanol (Zorka Pharma, Šabac, Serbia), *n*-hexane, toluene, cyclohexane, 2,2-diphenyl-1-picrylhydrazyl (DPPH) (Sigma Chemical, St. Louis, MO, USA), methanol, sodium carbonate (Zorka Pharma, Šabac, Serbia), Folin–Ciocalteu reagent, and gallic acid (97%) (Merck, Darmstadt, Germany) were used.

### 2.2. Plant Material

Camelina seeds (*Camelina sativa* L.), two spring cultivars, NS Slatka and NS Zlatka (developed at the Institute of Field and Vegetable Crops, Novi Sad, Serbia), were dried to a moisture content of 5.94% (m/m) and 6.74% (m/m), respectively. The seeds of both genotypes were ground in an electric mill to an average particle size of 0.5 mm.

### 2.3. Oil Extraction from Camelina Seeds

The oil isolation from camelina seeds of both genotypes (NS Zlatka and NS Slatka) was carried out using cold-pressing and solvent extraction techniques. The cold-pressed oil was obtained by pressing the cleaned and dried camelina seeds on a screw press (Ulimac Machine, Ancara, Turkey) with an electric motor (power of 1.5 kW and capacity of 5–45 kg/h). The solvent extraction of oil was performed using a Soxhlet extractor and *n*-hexane as a solvent of choice. The ground camelina seeds (100 g) were packed in a bag made of filter paper and inserted into the extraction chamber. The plant material was treated with 1 L of *n*-hexane heated at the boiling temperature for 4 h. The oil yield (Y) was calculated according to Equation (1).

(1) $$Y\left(\%\right)=\frac{m_{o}}{m_{im}}\times 100$$

where m_o_ is a mass of obtained oil, and m_im_ is an initial mass of dry plant material.

### 2.4. Physico-Chemical Properties

The physico-chemical characteristics, such as the moisture content, density, refractive index (Abbe refractometer AR3D, Krüss Optronic, Hamburg, Germany), viscosity (Fungilab viscometers, Visco basic plus, Hapog, New York, NY, USA), and the pH value (HI 9321, Hanna instruments, Lisbon, Portugal) were also analyzed in the oil samples. The viscosity of oil was defined at a speed rate of 60 rpm using the SP–R3 spindle. The pH value of the samples was measured at 22 ± 2 °C. The saponification number was analyzed based on the standard method [27].

### 2.5. Oxidative Stability

Peroxide value (PV) was determined using the iodometric (visual) endpoint method, in accordance with ISO standard 3960:2017 [28]. The *p*-Anisidine value (AnV) was determined according to ISO method 6885:2016 [29], spectrophotometrically, in quartz cuvettes at 350 nm, and using a blank sample. The total oxidation value (TOTOX) was calculated as twice the peroxide value plus the p-anisidine value. The acid value (AV) was determined according to ISO method 660:2009 [30]; the results were expressed as mg KOH/g. The oxidative stability of analyzed camelina oils was determined with a Rancimat apparatus from Metrohm, Switzerland, according to ISO method 6886:2016 [31], using a constant air flow (20 L/h) at 100 °C.

The specific extinctions at λ = 232 and λ = 268 nm (K_232_ and K_268_, respectively) of a 1% oil solution in cyclohexane were determined by measuring absorbance on a Varian Cary-100 UV–Vis spectrophotometer (Mulgrave, Victoria, Australia). Scanning with a resolution of λ = 1 nm was carried out in 1 × 1 cm quartz cuvettes at room temperature. Otherwise, the extinction coefficients at *λ* = 232 nm and λ = 268 nm indicate the presence of conjugated dienes and conjugated trienes, respectively. The oxidative stability of the oils was assessed following their exposure to conditions specified by the Schaal oven test. The oil sample of 50 mL was poured into an open glass container and heated at 63 ± 2 °C for 96 h. After that, 1 g of fresh or tempered oil samples was dissolved in 100 mL of cyclohexane. The absorbance of the samples was measured at λ = 232 nm and *λ* = 268 nm. The R–value, as a reliable indicator of oil quality, was calculated according to Equation (2).

(2) $$R-value=\frac{A_{232\;nm}^{1\%}}{A_{268\;nm}^{1\%}}$$

### 2.6. Oil Composition

#### 2.6.1. Analysis of Fatty Acids

To evaluate the fatty acid content of the camelina oil, fatty acid methyl esters were prepared using the SRPS EN ISO method [32]. The fatty acid composition was analyzed using gas chromatography (7890A, Agilent Technologies, Santa Clara, CA, USA) equipped with a flame ionization detector and fitted with a capillary column SP–2560 (100 m × 0.25 mm, d = 0.20 µm). Helium was used as the carrier gas. The initial column temperature of 140 °C was kept constant for 3 min, then raised to 220 °C (at a rate of 3 °C/min) and held for 5 min. Finally, the column temperature was increased to 240 °C (at a rate of 2 °C/min) and held for 10 min. The injector and detector temperatures were set to 250 °C. The individual peaks were identified by comparison of their retention times with the standard mixture of 37 FAME methyl esters. The results were expressed as the weight percent of the total fatty acid content.

The calculated oxidizability value (COX) of the oils was determined using Equation (3).

(3) $$COX=\frac{C18:1+10.3\times C18:2+21.6\times C18:3}{100}$$

The atherogenic index (AI) and thrombogenic index (TI) were evaluated as follows (Equations (4) and (5), respectively):

(4) $$AI=\frac{4\times C14:0+C16:0}{\sum MUFA+\sum \omega -3+\sum \omega -6}$$

(5) $$TI=\frac{C14:0+C16:0+C18:0}{0.5\times MUFA+0.5\times \sum \omega -6+3\times \sum \omega -3+\frac{\omega \;-\;3}{\omega \;-\;6}}$$

The ratio of hypocholesterolemic to hypercholesterolemic FA (HH) was calculated using Equation (6).

(6) $$HH=\frac{C18:1+C18:2+C18:3}{C14:0+C16:0}$$

#### 2.6.2. Tocopherol Content

The tocopherol content of the oil samples was analyzed according to Rabrenović et al. [33], using high pressure liquid chromatography (Waters Corporation, Milford, MA, USA). Tocopherols were identified on the basis of retention times determined for α-tocopherol, β-tocopherol, and γ-tocopherol separately, and their contents were estimated by external calibration curves.

#### 2.6.3. Total Carotenoid Content

The total carotenoid content was determined using the standard procedure [34], by measuring the absorbance of the oil dissolved in cyclohexane at λ = 445 nm. The content was calculated according to Equation (7) and expressed as milligrams of β-carotene per kilogram of the oil.

(7) $$\beta -carotene\left(\frac{mg}{kg}\right)=383\times \frac{E}{P\times C}$$

where E is the difference in absorbance between oil and cyclohexane samples, P is the beam path (cm), and C is the oil concentration (g/100 mL).

#### 2.6.4. Total Phenol Content

The total phenol content was determined according to the previously reported procedure with slight modifications [35]. The samples were prepared according to a procedure as follows: 3 g of the oil sample was dissolved in 15 mL of *n*-hexane. After that, the liquid-liquid extraction of antioxidants from the oil sample was carried out three times with 5 mL of methanol for 2 min. The extracts were left overnight and then “washed” with 25 mL of *n*-hexane. Exactly 0.1 mL of methanol fraction was treated with 1 mL of tenfold diluted Folin–Ciocalteu reagent in distilled water. In this sample, 1 mL of sodium carbonate solution (7%, *m/v*) was added and incubated for 90 min. The absorbance was measured at λ = 765 nm compared to the distilled water. The total antioxidant content was expressed in milligrams of gallic acid equivalents per kilogram of oil. A blank solution was also prepared in the same way, using an equivalent amount of methanol instead of the extract.

#### 2.6.5. Antioxidant Capacity Measured by DPPH

The antioxidant capacity of the extracted and cold-pressed oils was determined using the DPPH assay [36]. Various concentrations of oils were prepared in toluene from stock solutions (1 g of oil dissolved in 10 mL of toluene). The samples were vigorously mixed for 20 s with 1 mL of DPPH radical solution in toluene at a concentration of 3 × 10^–4^ mol/L. The sample was kept in the dark at room temperature for 30 min before measurements. Absorbance measurements of the samples were taken at λ_max_ = 517 nm, using toluene as the reference. Under the same conditions, the absorbance of the negative control sample was determined. This sample was previously prepared by diluting 1 mL of toluene solution of DPPH radicals with 2.5 mL of toluene. The DPPH radical inhibition (I_DPPH_) expressed in percentage was calculated according to Equation (8).

(8) $$I_{DPPH}\left(\%\right)=\frac{A_{C}-A_{S}}{A_{C}}\times 100$$

where A_S_ is the absorbance of the sample treated with DPPH radical solution, and A_C_ is the absorbance of the negative control solution.

The antioxidant capacity of the oil samples was evaluated based on the half-maximal inhibitory concentration (IC_50_ value), which represents the oil concentration necessary to inhibit 50% of the initial DPPH radical concentration. This value of all analyzed samples was obtained by interpolation.

### 2.7. Statistical Analysis

The results of the chemical analyses were analyzed statistically using IBM SPSS Statistics 25 (IBM, Chicago, IL, USA). Tukey’s multiple comparison procedure was used to compare the obtained results to each other. Identical letters in rows or columns denote the lack of differences at a significance level α = 0.05.

## 3. Results and Discussion

### 3.1. Physico-Chemical Properties of Camelina Seed Oils

Analysis of physico-chemical parameters is a basic indicator in the research of nutritional, biological, and sensory properties of oils. The values of each parameter of camelina seed oils of genotypes NS Zlatka and NS Slatk, obtained by the solvent extraction and cold-pressing techniques are shown in Table 1.

Camelina seed oil yield ranged from 26.0 to 30.0%, aligning with values reported in other studies for both spring and winter cultivars of camelina seed [3]. Statistical analysis revealed a significant difference in oil yield between the two genotypes, with the NS Slatka genotype producing a higher yield than the NS Zlatka genotype, regardless of the extraction method. Interestingly, cold-pressed oils showed yields comparable to those obtained through solvent extraction, which was unexpected since solvent extraction is generally considered superior regarding yield [16]. According to Mwithiga and Moriasi [37], oil yields in cold-pressing increased linearly with longer pressing durations and higher pressures. This may explain the comparable yields between the extraction methods observed in our study. The higher oil yield from NS Slatka camelina seeds highlights the potential of this genotype for future industrial oil production.

The moisture content in all camelina seed oil samples was consistently below 0.2%. A significant difference (*p* < 0.05) was observed between cold-pressed NS Slatka oil, which had the highest moisture content, and cold-pressed NS Zlatka oil, which had the lowest moisture content. However, the oil extraction method did not significantly affect moisture content (*p* > 0.05). The moisture levels in the analyzed oils were comparable to those reported in a previous study [11]. Moisture content is crucial because it affects subsequent processing and storage conditions. Higher moisture content can intensify oil aging and oxidation, promote the formation of acidic compounds, and support the growth of microorganisms. Therefore, maintaining low moisture content in oils is essential, and storage conditions should be controlled to prevent increases. Given the low moisture content observed in the oils, they are expected to remain stable over an extended period if stored properly.

The solvent-extracted oils exhibited a lower density compared to the cold-pressed oils (Table 1). The values of this parameter are consistent with the literature data for cold-pressed oils [22], while they are lower compared to those for solvent-extracted oils [25]. The density of the oil at room temperature varies depending on the extraction method. The higher density of cold-pressed oils can be attributed to the presence of natural compounds, seed impurities, and debris, along with proteins, phospholipids, vitamins, antioxidants, and sterols that are retained in the oil after cold-pressing [38].

As shown in Table 1, the refractive index was not significantly different (*p* > 0.05) between the analyzed oils, with values ranging from 1.477 to 1.482. The results are comparable to those reported by Piravi-vanak et al. [22]. The value of this parameter depends on various factors, including the length of the fatty acid chain, molecular weight, degree of conjugation, and degree of unsaturation.

All tested camelina oils had similar viscosities (around 50 mPa·s), indicating that neither the extraction method nor the camelina seed genotype influenced oil viscosity (*p* > 0.05). Oils generally behave as Newtonian fluids, with SFA content making a significant contribution to viscosity. All edible oils consist of triglycerides with various fatty acids that differ depending on the length of the chain, the degree and position of saturation, as well as the geometry of the double bond in the hydrocarbon chain. Longer chains and more double bonds generally lead to higher viscosity [39].

The solvent-extracted oils of both genotypes had a slightly higher pH value (around pH 8) compared to cold-pressed oils (around pH 7). In this case, the extraction method of oil and camelina seed genotype had a significant effect (*p* < 0.05) on the pH value of the oil. The cold-pressing technique most likely yields a higher percentage of components of a more acidic character, which are responsible for the lower pH value of the oil.

The oil extraction significantly affected the saponification number, which ranged between 28.06 and 58.90 mg KOH/g oil. The cold-pressed oil of the NS Zlatka genotype had saponification number around 50% lower than that observed for solvent-extracted NS Zlatka oil. In camelina seed oil from the NS Slatka genotype, the extraction had no significant effect (*p* > 0.05) on the saponification number. However, the saponification numbers for camelina seed oil from the NS Slatka genotype were lower than those for the NS Zlatka genotype obtained by solvent extraction. The values of the saponification number presented in this study were lower in comparison to those available in the literature [22]. Differences in saponification number values are likely due to variations in the chemical composition of the tested oils, which are influenced by the oilseed variety, growing location, and processing methods.

### 3.2. Oxidative Stability of Camelina Seed Oils

The intake of lipid oxidation products can pose serious health risks to consumers because they react with proteins, DNA, and phospholipids, contributing to chronic conditions like inflammation, cancer, and cardiovascular diseases [40]. The parameters, including PV, AnV, TOTOX, AV, and IP, determined by a Rancimat at 100 °C were used to evaluate the oxidative stability of the camelina oils (Table 2).

The PV of the tested camelina oils ranged from 1.32 to 1.93 meq O_2_/kg. The only significant difference (*p* > 0.05) was observed between solvent-extracted camelina seed oil from the NS Zlatka genotype, which had the lowest PV value, and cold-pressed camelina seed oil from the NS Slatka genotype, which had the highest PV value. Similar results of PV were obtained by Piravi-vanak et al. [22] for oil from camelina seed grown in four different temperate regions in Iran, while lower than that reported by Symoniuk et al. [41] for fresh cold-pressed camelina oil from Poland. Variations in the oxidation state of oils can be attributed to differences in the variety and quality of camelina seeds, breeding region, processing technology applied, and storage conditions of the final product [20]. PV is a measure of the amount of primary oxidation products (primarily hydroperoxides) present in the oil. It reflects the extent of oxidative rancidity, which can negatively impact taste, aroma, and nutritional quality. Oils high in PUFAs, like camelina oil, are generally more prone to oxidation. The tested camelina oils had low PV values, indicating they were either fresh or in the early stages of oxidation. Importantly, these values remained below the maximum threshold for cold-pressed and virgin oils (<15 meq O_2_/kg oil) [42], confirming their suitability in terms of oxidative stability.

The evaluated camelina seed oils exhibited AnV values ranging from 0.08 to 0.39. Camelina oil from the NS Slatka genotype showed lower AnV values compared to the NS Zlatka genotype, regardless of the oil extraction method. This suggests that the varietal characteristics of camelina seeds can influence AnV values. The obtained results are consistent with those reported by Momot et al. [19], Ratusz et al. [23], and Czaplicki et al. [43]. AnV measures the level of secondary oxidation products, such as aldehydes, ketones, hydrocarbons, alcohols, esters, and acids, formed from the breakdown of primary oxidation products. The low AnV of the tested oils suggests that primary oxidation products have not significantly degraded, indicating that the oils are safe for consumption concerning oxidation and hydrolysis products.

The calculated TOTOX value, as a measure of the total oxidation profile of oils, for the tested camelina seed oils ranged from 2.91 to 4.00. Similar to PV, the lowest TOTOX value showed solvent-extracted oil from the NS Zlatka genotype, while the cold-pressed oil from NS Slatka had the highest TOTOX value. Other studies reported similar values of TOTOX [11,22]. According to Bojanowska and Lamarska [44], a TOTOX value of 10 is the threshold for determining acceptable quality in edible oils, with values above this threshold indicating significant oxidation and reduced quality. In this study, all examined oils had TOTOX values below 10, demonstrating that they maintained good quality.

The AV of the tested camelina seed oils was low and ranged from 1.02 to 1.70 mg KOH/g, being consistent with those reported in other studies [20,45]. When comparing tested genotypes, NS Slatka camelina oil had lower (*p* < 0.05) AV values in comparison to NS Zlatka camelina oil. The solvent extraction method resulted in lower (*p* > 0.05) AV for camelina seed oils from both tested genotypes compared to the cold-pressing method. AV determines the amount of free fatty acids released from the oil’s triacylglycerol molecules during processing and storage. A higher AV in cold-pressed oils indicates a greater degree of hydrolytic degradation, potentially leading to rancidity and reduced oil quality. However, the results of this study contrast with findings by others [46,47], who reported higher AVs for solvent-extracted lemon seed and chia seed oils compared to cold-pressed oils. This discrepancy can be attributed to the minimal processing involved in cold pressing, which preserves enzymes that break down triacylglycerols into free fatty acids and retains natural impurities that facilitate hydrolysis. In contrast, solvent extraction applies heat, which inactivates these enzymes and removes impurities, leading to a lower acid value. Nevertheless, all tested oils in this study had AV values within the acceptable limit of 4.0 mg KOH/g for cold-pressed oils [42], confirming compliance with quality standards.

The oxidative stability of the tested camelina seed oils was also evaluated by the Rancimat method and expressed as the induction period (IP) at 100 °C. It can be observed in Table 2 that the analyzed camelina seed oils had low oxidative stability (IP = 4.19–4.72 h). For both camelina seed genotypes, solvent extracted oils showed higher IP values than cold-pressed oils. Solvent-extracted camelina oil of the NS Slatka genotype exhibited a significantly higher (*p* < 0.05) IP value compared to oil of the NS Zlatka genotype. A similar trend was observed in the cold-pressed oils, although the difference in IP between the two genotypes was not statistically significant (*p* > 0.05). A similar oxidation stability of camelina oils, determined under the same measurement conditions, has been previously reported [20,45]. The chemical composition of the oil has a significant impact on its oxidative stability. Oils high in PUFAs, particularly α-linolenic acid, which contains three double bonds in its molecular structure, are unstable and more likely to undergo oxidative changes. Given that camelina oil contains approximately 35% α-linolenic acid, the low IP was not surprising. According to Symoniuk et al. [41], linseed oil, which contains a higher level of α-linolenic acid (around 55%), exhibited the lowest oxidative stability, with an IP of 3.37 h. In the same study, black cumin oil and pumpkin oil, which are deficient in α-linolenic acid, demonstrated the highest oxidative stability at a temperature of 100 °C, with IPs of 38.34 and 22.45 h, respectively. Higher oxidative stability in solvent extracted oils compared to cold-pressed oils has been previously reported for walnut oil [48] and apricot seed oil [49]. As already mentioned, solvent extraction effectively removes free fatty acids, residual moisture, and other impurities that can promote oxidation. On the other hand, cold-pressing retains more natural compounds and impurities, which may be more susceptible to oxidative processes. It has been observed that adding 0.2% rosemary extract to camelina oil improved oxidative stability, confirmed by a higher IP [50].

The assessment of the oil quality, in terms of secondary oxidation changes (enzymatic or chemical), that took place during its isolation was carried out by spectrophotometric analysis. By measuring the absorbance at λ = 232 nm, the content of conjugated dienes as primary products of linoleic acid oxidation can be determined in the oil. The absorbance at λ = 268 nm indicated the content of unsaturated carbonyl compounds and conjugated trienes as secondary oxidation products. The absorbances at *λ* = 232 nm and λ = 268 nm reflect the oil’s oxidative status, with higher values indicating a greater presence of oxidation compounds in the samples. Also, a good metric for evaluating oil quality is the R–value, representing the ratio of conjugated dienes and conjugated trienes. A higher R–value indicates better oil quality. As can be seen, the difference in the content of conjugated dienes in fresh oil samples was very small (Table 3).

Oil extraction method and camelina seed genotype did not have an effect on K_232_ (*p* > 0.05). Regarding conjugated trienes, the solvent-extracted oils had a higher content of about 27% compared to the cold-pressed oils. The cold-pressed oils had a higher R–value (K_232_/K_268_ ratio), indicating a better oil quality compared to the solvent-isolated oils. In the literature, there is currently no standardized limit value for K_232_ and K_268_ for the cold-pressed oils. For this reason, it is important to refer to EU Commission Regulation 2568/91 [51] concerning extra virgin olive oil. According to this Regulation, the K232 value should not be higher than 2.50, and the K_268_ value should not be higher than 0.22. Concerning the content of conjugated dienes and trienes in all analyzed oils, the limit values prescribed by the EU Regulation were exceeded. In the cold-pressed oil of camelina seed from Poland [20], the K_232_ and K_268_ values were 1.76 and 0.26, respectively, which is in accordance with the values prescribed by the EU Regulation.

The results of the Schaal oven test for the analyzed oils are given in Table 3. Heating at 63 °C for 96 h did not significantly (*p* > 0.05) affect the K_232_ values of any of the analyzed oils. However, cold-pressed oils exhibited lower K_268_ values and higher R–values compared to solvent-extracted oils. This finding is inconsistent with the results obtained from the Rancimat test, which indicated superior quality in solvent-extracted oils. These observations suggest that cold-pressed oils demonstrate greater resistance to oxidative changes at moderate temperatures (63 °C) than at elevated temperatures (100 °C). In cold-pressed oils, the NS Zlatka genotype showed a higher K_268_ and lower R–value compared to the NS Slatka genotype.

### 3.3. Composition of Camelina Seed Oils

#### 3.3.1. Fatty Acid Profile and Nutrient Indices of Camelina Seed Oils

The evaluation of the fatty acid profile in edible oils is an important indicator for assessing their quality in terms of nutritional value, oxidative stability, and authentication purposes [25]. In Table 4, the fatty acid composition of the investigated camelina oils is presented.

The evaluated camelina oils exhibited a highly favorable fatty acid composition, with around 90% comprised of unsaturated acids, while SFAs constituted only around 10%. Comparable results have been reported by others [11,41,45,51]. Palmitic (C16:0) and stearic (C18:0) acids were the predominant SFAs in the oils examined, and their proportions were not affected (*p* > 0.05) by the oil extraction technique or camelina seed genotype. Similar results for palmitic and stearic acids in camelina oils were reported by Gawrysiak-Witulska et al. [52] and Piskernik et al. [53]. Solvent-extracted camelina oils exhibited higher levels of total SFAs compared to cold-pressed camelina oils across both camelina seed genotypes. It has been documented that elevated dietary intake of SFAs has a detrimental impact on the human body because they may increase the risks of diabetes, cardiovascular diseases, metabolic syndrome, heart failure, and mortality. Thus, it is recommended to limit the consumption of oils high in SFAs to maintain a healthy lifestyle [11]. The most dominant MUFAs in camelina oils were gondoic acid (C20:1n9) and oleic acid (C18:1n9c). Other researchers have shown similar levels of these fatty acids [11,19]. Higher percentages of oleic acid (19.78–20.47%) were reported by Piravi-vanak et al. [22] and Ergönül and Özbek [24], who evaluated camelina oil from seeds grown in Turkey and Iran, respectively. The variations in the content of these fatty acids can be ascribed to the impact of the growing conditions and different varieties. The total content of MUFAs was around 32% and did not differ (*p* > 0.05) between the oil samples. Erucic acid (C22:1n9) was also detected in camelina oil, with its content ranging from 2.91% to 3.18%. Comparable content of erucic acid has been obtained by Ratusz et al. [20], who studied camelina oil from seeds grown in Poland. Both solvent-extracted and cold-pressed camelina oil from NS Zlatka showed significantly (*p* < 0.05) higher levels of erucic acid than that of the NS Slatka genotype, indicating varietal characteristics. Erucic acid is a naturally occurring plant toxin found in the oilseeds that belong to the Brassicaceae family. High dietary intake of erucic acid can lead to myocardial lipidosis, a heart condition characterized by fat accumulation in heart tissues, and may also produce toxic effects. Based on that, the EU Commission established a maximum threshold for erucic acid in vegetable oils and fats intended for final consumer or use as an ingredient in food at 20 mg/kg, while for camelina oil the maximum limit is 50 g/kg of total fatty acids [54]. Additionally, Wallace et al. [55] established a tolerable daily intake of erucic acid of 7 mg/kg body weight per day. However, the level of erucic acid in tested camelina oils was lower than the maximum limit. The total percentage of PUFAs in the examined camelina oils ranged from 57.19% to 58.88% and was not affected (*p* > 0.05) by the oil extraction technique or camelina seed genotype. The previous studies evidenced that the extraction method (solvent extraction and cold-pressing method) had no significant effect on the fatty acid composition of oils from apricot seeds and chia seeds [46,49]. α-Linolenic acid (C18:3n3) was the most abundant PUFA in camelina oils (36.68–39.25%). Camelina oil is the second most abundant plant-based source of α-linolenic acid, surpassed only by linseed oil (up to 60%). Common vegetable oils are a poor source of α-linolenic acid. This fatty acid, which belongs to the group of ω-3 fatty acids, is considered essential because it plays key roles in various biological processes, making it vital for the body’s proper functioning. Linoleic acid (C18:2n6), belonging to the group of ω-6 fatty acids, is also considered an essential fatty acid, and its percentage in camelina oils ranged from 15.83% to 16.12%. Since the human body cannot produce essential fatty acids independently, they must be obtained from diet. The determined contents of these acids are comparable to those presented in other works [20], whereas Ergönül and Özbek [24] reported higher content of linoleic acid (21.25–24.05%) but lower percentages of α-linolenic acid (22.31–26.57%). It is well known that PUFAs may help prevent a range of conditions, including coronary heart disease, atherosclerosis, thrombotic diseases, diabetes, inflammatory diseases, cancer, Crohn’s disease, etc. [56]. On the other hand, PUFAs are sensitive to the effects of air, light, and high temperatures due to the presence of two or more double bonds in the basic hydrocarbon chain. For these reasons, their heat treatment is not recommended. Due to its unique fatty acid profile, camelina oil could be suitable for direct consumption, including use in the enrichment of various food products with ω-3 fatty acids, such as margarine, salad dressings, mayonnaise, and ice cream. Its potential nutraceutical benefits suggest its use in the development of functional foods and nutraceuticals aimed at promoting health [57]. Furthermore, the high ω-3 fatty acids of camelina oil make it a valuable ingredient in cosmetics, with dermatological benefits that support its use in cosmetic oils, skin lotions, serums and creams [24]. Owing to its high content of PUFAs and ω-3 fatty acids, camelina oil can effectively serve as a substitute for fish oil in aquaculture feed. Additionally, it can be included into the diets of broilers, laying hens, and dairy cows to enrich animal-derived products, such as meat, eggs, and milk, with ω-3 fatty acids, enhancing their nutritional profile for human consumption [6].

A proper balance between ω-6 and ω-3 fatty acids is crucial for supporting various physiological functions and achieving optimal health. The recommended dietary ratio is approximately 4:1 or lower. In the investigated camelina oils, the ω-6/ω-3 ratio was in the range of 0.44–0.49, indicating the high nutritional value of the camelina oils. This ratio is markedly superior to that typically found in commonly used vegetable oils, such as pumpkin, sunflower, and rapeseed oil [11,41]. In addition, the COX related to the oxidative stability of oil, as well as nutritional quality indices such as AI, TI, and HH, were calculated based on the fatty acid composition of the camelina oils. The COX refers to a measure used to assess the susceptibility of fatty acids to oxidation. The COX values of the evaluated camelina seed oils varied between 9.78 and 10.25, which is similar to those found in previous studies [20]. A higher COX value indicated that camelina oil is more prone to oxidative deterioration, which can affect the quality, flavor, and nutritional value of oils. Low values of AI (0.06–0.07) and TI (0.05–0.06) were observed for the analyzed camelina oils and did not significantly differ (*p* > 0.05) between oils. The obtained results for AI and TI are similar to those presented by other authors [11,20]. The AI indicates the potential of fatty acids to contribute to atherosclerosis, whereas TI assesses the thrombogenic potential of fatty acids in relation to blood clot formation and the risk of thrombosis [56]. From a nutritional perspective, opting for oils with low values of both AI and TI is preferable for a healthy diet. The HH of the camelina seed oils was in the range of 11.54–12.75, which was in accordance with previously published results [11,20]. The lowest value (*p* < 0.05) of HH was found in solvent-extracted camelina seed oil from the NS Zlatka genotype, while the highest value (*p* < 0.05) was found in the cold-pressed oil from the NS Slatka genotype. The h/H ratio is an index used to describe the relative effects of different fatty acids on cholesterol metabolism. A higher h/H ratio is considered more beneficial for human health [58]. Therefore, incorporating camelina oil into the diet, characterized by its abundance of unsaturated fatty acids, a balanced ω-6 to ω-3 ratio, low AI and TI values, and a relatively high HH ratio, has the potential to lower the occurrence of numerous chronic diseases.

#### 3.3.2. Tocopherol Content of Camelina Seed Oils

The camelina oils were characterized by a considerable content of tocopherols (Table 5).

The content of α-tocopherols in the evaluated camelina oils ranged from 10.8 to 26.8 mg/kg. Cold-pressed oils contained approximately 1.5 to 2 times more α-tocopherol than solvent-extracted oils. The β-tocopherol content in all samples was below the detection limit. The γ-tocopherol levels varied between 184.3 and 547.4 mg/kg, with cold-pressed oils showing significantly higher concentrations (*p* < 0.05) than solvent-extracted oils. Specifically, γ-tocopherol content was up to 2.7 times greater in cold-pressed oils than in solvent-extracted oils. Cold-pressed oil from the NS Slatka genotype demonstrated higher γ-tocopherol content compared to cold-pressed oil from the NS Zlatka genotype. Similar to our findings, previous studies have reported comparable concentrations of α-tocopherols in cold-pressed camelina oils [11,22]. The amount of β-tocopherol in the analyzed oils agrees with Rabiej-Kozioł et al. [11]. The γ-tocopherol content in the cold-pressed camelina oils was consistent with the levels observed in camelina oils grown in Poland [43,52]; however, higher γ-tocopherol content has been observed in camelina oils cultivated in Turkey [24], Iran [22], and Slovenia [59]. This indicates that tocopherol content can vary depending on the growing region. Similar to our observations, Uluata [49] showed that cold-pressed apricot seed oil had a nearly 1.5-fold higher amount of α-, γ-, and total tocopherols than the solvent-extracted counterparts. Additionally, the amount of α-tocopherol was greater in the cold-pressed lemon seed oils than that of the solvent-extracted [47]. It is evident that the oil extraction method had a substantial impact on tocopherol content. The cold-pressing method extracts oil by mechanically crushing plant material at low temperatures, without the use of chemical solvents. This process preserves heat-sensitive compounds, such as tocopherols. Consequently, cold-pressed oil retains a composition that is closer to its natural state. In contrast, solvent extraction employs chemicals and high temperature, which can alter the oil’s composition by selectively removing or degrading some natural compounds. Tocopherols, as potent antioxidants, are crucial for protecting PUFAs in the oils from peroxidation, thereby enhancing oil stability and nutritional value [11]. The antioxidant properties also offer preventive and therapeutic roles in diseases, such as Alzheimer’s and cancer, by trapping free radicals and chelating metal ions [43]. The antioxidant potential of tocopherol isomers is influenced by their concentrations in the bulk oils. Research indicates that the most effective antioxidant potential of γ-tocopherol, without exhibiting prooxidant effects, occurs at concentrations between 250 and 500 mg/kg [60]. The γ-tocopherol levels in the tested oils fell within this optimal range.

#### 3.3.3. Total Carotenoid Content of Camelina Seed Oils

Another group of bioactive compounds analyzed in the oil are carotenoids. Their total content in the oil, expressed as β-carotene content (mg/kg oil), is shown in Table 5. The results of this study showed that camelina seed oils are rich in carotenoids, especially those obtained by solvent extraction (118.96 mg/kg and 132.52 mg/kg for camelina seed oils of the NS Zlatka and NS Slatka genotypes, respectively). Camelina seed genotype did not have a significant influence (*p* > 0.05) on the β-carotene content in the oils. The content of β-carotene determined in the oils of other camelina seed genotypes was similar [3,45]. Higher β-carotene content in solvent-extracted oils is likely due to differences in extraction conditions. Specifically, *n*-hexane, a non-polar solvent, effectively dissolves and extracts lipophilic compounds such as carotenoids, including β-carotene, leading to a greater concentration of β-carotene in the oils. The presence of carotenoids in the oil is significant, not only because of its provitamin role but also because of its positive impact on human health as an antioxidant. Taking into account that the human body’s daily need for vitamin A is between 700 and 900 μg [61], camelina seed oil can be used as a food supplement or for the development of dietary supplements that will meet vitamin A needs.

#### 3.3.4. Total Phenol Content of Camelina Seed Oils

Oil-rich seeds are known to contain many antioxidants, but only a small fraction of them make it into the oil after pressing or extraction. Having this in mind, the content of hydrophilic antioxidants is not expected to be the same as in the seeds themselves. As can be seen in Table 5, the total phenol content ranged between 7.3 and 41.1 mg GAE/100 g, which was much higher than total phenol content in camelina oil reported by Ergönül and Özbek [24], while lower than that reported by Symoniuk et al. [41]. The oil extraction method and genotype had a significant effect (*p* < 0.05) on total phenol content. A higher total phenolic content was found in camelina oils of the NS Slatka genotype compared to the NS Zlatka genotype, regardless of the oil extraction method. The cold-pressed camelina oil of the NS Slatka genotype had a total phenol content about 25% lower compared to the solvent-extracted NS Slatka camelina oil. The reduction in total phenol content between the solvent-extracted and cold-pressed oil for the NS Zlatka genotype was about 72%. Based on the obtained results, it can be concluded that the solvent-extracted oils are richer in total phenols compared to the cold-pressed oils. It is known that extracting oil at higher temperatures results in enhanced release of phenolic compounds from the seeds [62].

#### 3.3.5. Antioxidant Capacity of Camelina Seed Oils

The antioxidant capacity of camelina seed oils was determined using the DPPH test. The IC_50_ values of the analyzed oils were obtained by interpolation and are shown in Table 6.

The cold-pressed oil of the genotype NS Slatka had the lowest IC_50_ value (9.50 mg/mL), which indicates that this oil has the best antioxidant capacity, measured by DPPH assay. A slightly weaker ability to trap free radicals was observed in the solvent-extracted oil of the NS Zlatka genotype. The highest IC_50_ showed solvent extracted NS Slatka and cold-pressed NS Zlatka camelina oils. The antioxidant capacity of oil is linked to its capacity to neutralize free radicals that initiate oxidation processes in fats. In cold-pressed oils, these free radicals primarily interact with phenolic compounds, carotenoids, tocopherols, tocotrienols, and specific sterols, helping to maintain the stability and quality of the oil [41]. The greater ability of cold-pressed NS Slatka oil to scavenge free radicals can be attributed to the highest content of γ-tocopherols and pronounced content of α-tocopherols observed in that oil. Although cold-pressed NS Zlatka oil had high γ-tocopherol content, it did not exhibit a lower IC_50_ value. Similarly, even though solvent-extracted oil of NS Slatka camelina seed genotype contained the highest total phenol levels and significant β-carotene content, it demonstrated weak free radical scavenging activity. Symoniuk et al. [41] observed that oils, even when rich in phenolic compounds, do not always display optimal antioxidative capacity. It should be emphasized that the antioxidant capacity of oils can be affected by several factors, including the type and concentrations of antioxidants present, as well as the presence of other substances, all of which play a crucial role in how well the oil can neutralize free radicals. In general, camelina seed oils of both genotypes are sources of natural antioxidants, which can make a certain contribution to the autooxidation stability of the oil.

## 4. Conclusions

This paper evaluated the effects of different oil extraction methods, cold pressing without heat employed and solvent extraction, on the physico-chemical parameters, oil composition, antioxidative capacity, and oxidative stability of camelina oils from two different camelina seed genotypes. The analysis of physico-chemical parameters showed that the oils obtained by different extraction methods had similar yields and were of satisfactory quality. Camelina seed oils were rich in polyunsaturated fatty acids (around 58%), of which α-linoleic acid was the most abundant (36.68–39.25%). High content of unsaturated fatty acids with a balanced ω-6 to ω-3 ratio, low AI and TI values, and a relatively high HH ratio raise the possibility of including camelina oils in the human diet. Cold-pressed oils exhibited a higher content of α- and γ-tocopherols and demonstrated greater oxidative stability at moderate temperatures, as evidenced by the Schaal oven test. However, their oxidative stability decreased at elevated temperatures (100 °C) compared to solvent-extracted oils. In contrast, solvent-extracted oils contained higher levels of β-carotene and exhibited superior resistance to high-temperature conditions. When comparing the nutritive quality of tested camelina seed genotypes, cold-pressed oil of the NS Slatka genotype demonstrated the highest content of γ-tocopherols, along with a significant amount of α-tocopherols and total phenols. Utilizing cold-pressing of camelina seeds to produce oil with high nutritional quality presents significant opportunities for application across diverse industries, including food, nutraceuticals, animal feed, and cosmetics, especially in applications that avoid thermal treatments. This approach not only enhances the product range but also promotes the use of natural and health-beneficial oils in various markets. Future investigations may focus on enhancing the oxidative stability and shelf life of cold-pressed oils through the inclusion of natural antioxidants.

## Figures and Tables

**Table 1:** Physico-chemical properties of camelina seed oil obtained by solvent extraction and cold-pressing.

Parameters	Solvent Extraction		Cold-Pressing	
	NS Zlatka	NS Slatka	NS Zlatka	NS Slatka
Oil yield (%)	26.7 ± 0.04 ^a^	29.7 ± 0.06 ^b^	26.0 ± 0.06 ^a^	30.0 ± 0.08 ^b^
Moisture content (%)	0.171 ± 0.02 ^ab^	0.149 ± 0.03 ^ab^	0.118 ± 0.01 ^a^	0.193 ± 0.02 ^b^
Density (g/mL)	0.8718 ± 0.05 ^a^	0.8253 ± 0.03 ^a^	0.9094 ± 0.04 ^b^	0.9150 ± 0.02 ^b^
Refractive index	1.479 ± 0.01 ^a^	1.482 ± 0.02 ^a^	1.477± 0.01 ^a^	1.481 ± 0.01 ^a^
Viscosity (mPa·s)	50.2 ± 2.29 ^a^	49.9 ± 2.21 ^a^	50.1 ± 2.49 ^a^	49.6 ± 2.18 ^a^
pH value	7.40 ± 0.03 ^c^	8.10 ± 0.04 ^d^	6.66 ± 0.02 ^a^	6.80 ± 0.02 ^b^
Saponification number (mg KOH/g)	58.90 ± 3.17 ^c^	42.09 ± 2.26 ^b^	28.06 ± 1.89 ^a^	47.70 ± 3.42 ^b^

Values are the mean of three replications ± standard deviation. ^a,b,c^—Letters that are the same within rows indicate no significant differences at a 0.05 significance level.

**Table 2:** Oxidative stability of camelina seed oils obtained by solvent extraction and cold-pressing.

Parameters	Solvent Extraction		Cold-Pressing	
	NS Zlatka	NS Slatka	NS Zlatka	NS Slatka
Peroxide value (meq O_2_/kg)	1.32 ± 0.21 ^a^	1.80 ± 0.06 ^ab^	1.60 ± 0.11 ^ab^	1.93 ± 0.09 ^b^
*p*-Anisidine value	0.27 ± 0.07 ^bc^	0.08 ± 0.01 ^a^	0.39 ± 0.04 ^c^	0.15 ± 0.05 ^ab^
TOTOX	2.91 ± 0.35 ^a^	3.68 ± 0.12 ^ab^	3.58 ± 0.18 ^ab^	4.00 ± 0.13 ^b^
Acid value (mg KOH/g)	1.40 ± 0.01 ^c^	1.02 ± 0.01 ^a^	1.70 ± 0.02 ^d^	1.12 ± 0.01 ^b^
Induction period at 100 °C	4.48 ± 0.05 ^b^	4.72 ± 0.04 ^c^	4.19 ± 0.05 ^a^	4.26 ± 0.06 ^a^

Values are the mean of three replications ± standard deviation. ^a,b,c,d^—Letters that are the same within rows indicate no significant differences at a 0.05 significance level.

**Table 3:** The extinction coefficient of fresh and tempered camelina seed oils at λ = 232 nm and λ = 268 nm obtained by solvent extraction and cold-pressing.

Parameters	Oil	Solvent Extraction		Cold-Pressing	
		NS Zlatka	NS Slatka	NS Zlatka	NS Slatka
K_232_	Fresh oil	3.5451 ± 0.08 ^a^	3.6450 ± 0.03 ^a^	3.5497 ± 0.06 ^a^	3.5246 ± 0.07 ^a^
	Tempered oil	3.5709 ± 0.07 ^a^	3.6488 ± 0.07 ^a^	3.5914 ± 0.04 ^a^	3.5733 ± 0.01 ^a^
K_268_	Fresh oil	3.6463 ± 0.04 ^b^	3.7459 ± 0.07 ^b^	2.6828 ± 0.06 ^a^	2.6661 ± 0.08 ^a^
	Tempered oil	3.6694 ± 0.06 ^c^	3.7467 ± 0.04 ^c^	2.6945 ± 0.01 ^a^	2.9085 ± 0.07 ^b^
R–value	Fresh oil	0.9722 ± 0.013 ^a^	0.9731 ± 0.010 _a_	1.3231 ± 0.007 ^b^	1.3220 ± 0.013 ^b^
	Tempered oil	0.9732 ± 0.003 ^a^	0.9739 ± 0.008 ^a^	1.3329 ± 0.001 ^c^	1.2286 ± 0.026 ^b^

Values are the mean of three replications ± standard deviation. ^a,b,c^—Letters that are the same within rows indicate no significant differences at a 0.05 significance level.

**Table 4:** Composition of fatty acids and nutrient indices in camelina seed oil samples obtained by solvent extraction and cold-pressing.

Fatty Acid (%)	Solvent Extraction		Cold-Pressing	
	NS Zlatka	NS Slatka	NS Zlatka	NS Slatka
C14:0	0.06 ± 0.01	0.05 ± 0.01	0.04 ± 0.01	0.05 ± 0.01
C16:0	5.75 ± 0.17	5.75 ± 0.15	5.39 ± 0.16	5.35 ± 0.15
C18:0	2.76 ± 0.20	2.58 ± 0.18	2.50 ± 0.17	2.48 ± 0.15
C20:0	1.43 ± 0.03	1.31 ± 0.21	1.30 ± 0.02	1.18 ± 0.01
ΣSFA	9.99 ± 0.07 ^d^	9.69 ± 0.05 ^c^	9.23 ± 0.06 ^b^	9.07 ± 0.04 ^a^
C16:1	0.09 ± 0.01	0.11 ± 0.03	0.10 ± 0.02	0.10 ± 0.03
C18:1n9c	13.59 ± 0.48	13.94 ± 0.51	13.40 ± 0.49	13.87 ± 0.51
C20:1n9	16.12 ± 0.69	15.72 ± 0.58	15.44 ± 0.52	15.17 ± 0.48
C22:1n9	3.03 ± 0.04 ^b^	2.91 ± 0.01 ^a^	3.18 ± 0.03 ^c^	2.91 ± 0.02 ^a^
ΣMUFA	32.82 ± 3.17	32.67 ± 3.09	32.12 ± 2.92	32.05 ± 2.79
C18:2n6c	16.71 ± 1.84	16.72 ± 1.88	15.89 ± 1.79	15.83 ± 1.77
C18:3n3	36.68 ± 3.44	37.96 ± 3.68	38.76 ± 3.87	39.25 ± 3.94
C20:2n6	1.99 ± 0.18 ^b^	1.17 ± 0.16 ^a^	2.11 ± 0.19 ^b^	2.03 ± 0.23 ^b^
C20:3n3	1.80 ± 0.01 ^a^	1.79 ± 0.02 ^a^	1.89 ± 0.01 ^b^	1.77 ± 0.01 ^a^
ΣPUFA	57.19 ± 2.61	57.63 ± 2.55	58.65 ± 2.46	58.88 ± 2.39
Σω-3	38.48 ± 2.26	39.75 ± 2.29	40.65 ± 2.34	41.02 ± 2.38
Σω-6	18.71 ± 0.17 ^b^	17.89 ± 0.12 ^a^	18.00 ± 0.03 ^a^	17.86 ± 0.17 ^a^
ω-6/ω-3	0.49 ± 0.02	0.45 ± 0.02	0.44 ± 0.02	0.44 ± 0.02
COX	9.78 ± 0.04	10.06 ± 0.24	10.14 ± 0.06	10.25 ± 0.05
AI	0.07 ± 0.00	0.07 ± 0.00	0.06 ± 0.00	0.06 ± 0.00
TI	0.06 ± 0.00	0.06 ± 0.00	0.05 ± 0.00	0.05 ± 0.00
HH	11.54 ± 0.03 ^a^	11.83 ± 0.45 ^ab^	12.54 ± 0.22 ^ab^	12.75 ± 0.22 ^b^

SFAs—saturated fatty acids; MUFAs—monounsaturated fatty acids; PUFAs—polyunsaturated fatty acids; COX—calculated oxidizability value; AI—atherogenic index; TI—thrombogenicity index; HH—ratio of hypocholesterolemic to hypercholesterolemic fatty acids; values are the mean of three replications ± standard deviation. ^a,b,c,d^—Letters that are the same within rows indicate no significant differences at a 0.05 significance level.

**Table 5:** Content of tocopherols, β-carotene, and total phenols of camelina seed oils obtained by solvent extraction and cold-pressing.

Parameters	Solvent Extraction		Cold-Pressing	
	NS Zlatka	NS Slatka	NS Zlatka	NS Slatka
α-tocopherol (mg/kg)	14.1 ± 1.34 ^a^	10.8 ± 0.28 ^a^	21.1 ± 0.99 ^b^	26.8 ± 2.33 ^b^
β-tocopherol (mg/kg)	<0.1	<0.1	<0.1	<0.1
γ-tocopherol (mg/kg)	184.3 ± 8.98 ^a^	198.2 ± 11.46 ^a^	385.1 ± 30.48 ^b^	547.4 ± 10.54 ^c^
β-carotene (mg/kg)	118.96 ± 10.9 ^b^	132.52 ± 12.4 ^b^	89.43 ± 6.01 ^a^	71.12 ± 5.28 ^a^
Total phenol content (mg GAE/100 g)	26.0 ± 0.02 ^b^	41.1 ± 0.02 ^d^	7.3 ± 0.02 ^a^	30.9 ± 0.02 ^c^

Values are the mean of three replications ± standard deviation. ^a,b,c,d^—Letters that are the same within rows indicate no significant differences at a 0.05 significance level.

**Table 6:** Antioxidant capacity of camelina seed oils obtained by solvent extraction and cold-pressing.

Antioxidant Activity	Solvent Extraction		Cold-Pressing	
	NS Zlatka	NS Slatka	NS Zlatka	NS Slatka
IC_50_ (mg/mL)	10.50 ± 0.07 ^b^	11.90 ± 0.04 ^c^	11.90 ± 0.08 ^c^	9.50 ± 0.06 ^a^
Concentration (mg/mL)	3.125–100	3.125–100	6.250–100	6.250–100

IC_50_—half of the maximum inhibition of free DPPH radicals; ^a,b,c^—Letters that are the same within rows indicate no significant differences at a 0.05 significance level.

## Data Availability

The original contributions presented in the study are included in the article, further inquiries can be directed to the corresponding author.
